# Doctor's Wills

**Published:** 1914-02-07

**Authors:** 


### Doctor's Wills.

Brigade-Surgeon Edward Footner, M.D., has left estate of the gross value of £17,263, of which £16,365 is net personalty.

Surgeon-Colonel John Richardson, I.M.S. (retired), who saw service in the Bhutan expedition of 1864-66, hon. physician to the King in 1903, has left net personalty £3,256.

Dr. Edward Austin Fox, M.D., of New Brighton, formerly of Warrington, consulting surgeon, and a life governor of the Warrington Infirmary, has left net personalty £28,908.

Dr. Frederick Dale, M.D., J.P., one of the hon. consulting surgeons at the Scarborough Hospital and Dispensary, has left estate valued at £29,521 gross, with net personalty £22,849.

